# Common errors and clinical guidelines for manual muscle testing: "the arm test" and other inaccurate procedures

**DOI:** 10.1186/1746-1340-16-16

**Published:** 2008-12-19

**Authors:** Walter H Schmitt, Scott C Cuthbert

**Affiliations:** 1 Chiropractic Health Center, 255 West Abriendo Avenue, Pueblo, CO 81004, USA

## Abstract

**Background:**

The manual muscle test (MMT) has been offered as a chiropractic assessment tool that may help diagnose neuromusculoskeletal dysfunction. We contend that due to the number of manipulative practitioners using this test as part of the assessment of patients, clinical guidelines for the MMT are required to heighten the accuracy in the use of this tool.

**Objective:**

To present essential operational definitions of the MMT for chiropractors and other clinicians that should improve the reliability of the MMT as a diagnostic test. Controversy about the usefulness and reliability of the MMT for chiropractic diagnosis is ongoing, and clinical guidelines about the MMT are needed to resolve confusion regarding the MMT as used in clinical practice as well as the evaluation of experimental evidence concerning its use.

**Discussion:**

We expect that the resistance to accept the MMT as a reliable and valid diagnostic tool will continue within some portions of the manipulative professions if clinical guidelines for the use of MMT methods are not established and accepted. Unreliable assessments of this method of diagnosis will continue when non-standard MMT research papers are considered representative of the methods used by properly trained clinicians.

**Conclusion:**

Practitioners who employ the MMT should use these clinical guidelines for improving their use of the MMT in their assessments of muscle dysfunction in patients with musculoskeletal pain.

## Background

Since Goodheart introduced applied kinesiology (AK) as a chiropractic clinical concept in 1964 [1], the use of manual muscle testing (MMT) has become widespread throughout the chiropractic profession and has spilled over into the medical, dental and other health professions as a mode of analysis of nervous system function [2-17]. The MMT is used in the chiropractic, orthopedic, neurological, medical, dental, homeopathic, and physical therapy arenas to assess locomotor system dysfunction and to determine a patient's progress during therapy.

In the words of the cliché, "Imitation is the sincerest form of flattery." In the practice of muscle testing, however, imitation has proven to be an embarcadero for inconsistency. All too many well-meaning clinical investigators observed the phenomena of muscles strengthening and weakening during standard AK and MMT procedures, and have embarked on a course of investigation using the tool of muscle testing without the benefit of formalized training and experience in standard MMT practices [18-22].

Similarly, in research efforts that evaluate the validity of claims from physicians who employ the MMT as part of their diagnostic and treatment programs, the MMT has been used in a large number of studies in a way that that does not reflect the methods used in clinical practice [18-22]. Critical evaluation of the quality of the research methodology employed in studies of chiropractic diagnostic methods, especially the MMT, are necessary but will be irrelevant to conclusions regarding the MMT and/or AK if the process examined relates poorly to the practice of MMT or AK [23]. For instance many of these papers investigate a false premise about AK, just as many of them employ the MMT improperly. A simplistic notion of universal effects of certain stimuli such as "tasting sugar makes one weak" or exact correspondence between single MMT results and certain pathological states are not valid in AK [24,25]. In clinical practice AK MMT is used as an adjunct rather than alternative to other standard diagnostic measures. Multiple AK MMTs are performed in series and parallel fashion before any diagnosis is ever made. The importance of correlating MMT findings with standard diagnostic procedures has been a fundamental rule of AK teachings from the beginning [1,14,15].

Two recent reviews by Haas et al. and Hall et al. [26,27] are examples of this. Regarding the chiropractic use of the MMT and of AK chiropractic technique, Haas lists seven papers as examples of "AK research" [18-22,24,25]. The studies chosen by Haas show poor reliability and outcomes indeed, but they also employ non-standard MMT and/or investigate methods of testing that the International College of Applied Kinesiology (I.C.A.K.) neither supports nor teaches [18-22,24,25]. More important to the present discussion, the methods of MMT employed in these studies ("the arm pull down test" was used in 3 of the studies) [18,20,21], were not the standardized MMT taught by the I.C.A.K. or the chiropractic colleges who now teach MMT methods to students.

In another review by Hall et al. [27] the inclusion criteria of the reviewed literature specifically excluded most of the professional research literature relevant to AK, because the AK methods of MMT did not meet their definition of "kinesiology" practice. The authors limited their search criteria to the "light muscle test" which was the authors' interpretation of the Touch for Health's system of two-finger pressure testing. Touch for Health was developed by an AK chiropractor as a simple offshoot of the AK chiropractic method that could be taught to the public and to this doctor's patients in weekend seminars. Hall's review confounded this system of MMT for laypeople with the chiropractic AK system of MMT based on the works of Kendall & Kendall. The authors confounded their judgment about the professional use of AK by entangling it with the "Specialised Kinesiologies" and "energy kinesiologies" of the Touch for Health offshoots. Many of the methods the authors describe as "Kinesiology" (which have nothing whatever to do with AK) even diverge dramatically from Touch for Health's original home health program, such as "astrological kinesiology" [28].

The pressing need for clinical guidelines regarding what is and what is not standardized MMT is obvious in the reviews of Haas and Hall et al [23].

These unfortunate circumstances, coupled with the enthusiasm generated when a method is discovered that can potentially justify otherwise empirical procedures, enhance the chance for intrusion into clinical investigations of "expectancy" and "operator prejudice." Operator prejudice is the specter that haunts clinical research and can invalidate thousands of clinical trials involving months or years of effort by a simple error in the researcher's evaluation of his investigative parameters.

The ideomotor effect (the unconscious and inadvertent cueing of desired responses) will also be prevented if examiners follow standardized protocols that specify patient and examiner position, the precise alignment of the muscle being tested, proper timing of the MMT, the direction of the resisting force applied to the patient, and the verbal instruction or demonstration to the patient [29]. The use of the MMT demands rigorous attention to every detail that might affect the accuracy of the test. The examiner must develop the ability to apply pressure or resistance in a manner that permits the subject to exert the optimal response – these factors are part of the science and the art of MMT.

In an effort to heighten the awareness of accuracy in muscle testing and increase the amount of reproducible new clinical material, this paper deals with some of the most common mistakes that have been adopted in the use of the MMT and how they are improperly performed and misinterpreted. Future clinical and research activity using the MMT should adhere to the principles described in this paper.

## Rationale – why MMT?

The technique of MMT began with Lovett in 1912 [2,13]. A system for grading the strength of postural muscles using the MMT for disability evaluation in polio and other neuromuscular diseases was presented by the Kendalls in 1936, with the first text based on this work published in 1949 [13].

Kendall and Kendall's second book was called *Posture and Pain *(1952), and it was already realized that the theoretical construct of the MMT should be expanded far beyond the "polio syndromes" that the MMT was originally designed to evaluate [12]. Using the detailed records from 12,000 cases they state, "The importance of muscle testing in cases of postural disorders cannot be over-emphasized."

Goodheart introduced this method of testing into the chiropractic profession in 1964, and he and the International College of Applied Kinesiology (I.C.A.K.) developed methods for treating the muscle inhibitions found using manual methods since that time [1,14].

The later works of Panjabi, Janda, Lewit, Jull, Sahrmann, Bergmark, Hammer and Liebenson have confirmed the findings of these earlier researchers, showing that muscles respond in predictable ways to pain, inflammation, and/or injury [12,30-36]. These researchers have also demonstrated that functional pathology of the muscle system is the most common clinical finding in pain patients presenting to chiropractors, osteopaths, neurologists, rheumatologists, orthopedists, and physical therapists. Yet this disorder of the muscle system is routinely ignored in the diagnosis and treatment of these patients.

The diagnosis of muscular imbalance with the MMT may offer clinicians a method for discovering where functional pathologies of the locomotor system exist and which ones are the most clinically significant. Methods for the objective evaluation of the effects of neuromuscular impairment and the measurement of changes in neuromuscular functioning must be developed in parallel with advances in therapy, and the MMT may be a tool for measuring this.

There is now evidence that *impaired strength *of specific muscles occurs in close relationship with the development of specific joint dysfunction, inflammation, or injury. The evidence shows that inflammation or injury specifically in the ankle [37], knee [38-40], lumbar spine [41-43], temporomandibular joint [44], and cervical spine [45-48] will produce *inhibited *muscles. These studies highlight the fact that the measurement of neuromuscular performance could be recognized as a fundamental contribution to restorative and rehabilitative treatment programs.

There is also evidence that there is an immediate effect upon the motor system (both locally and globally) after chiropractic manipulative therapy (CMT) [49-55]. Dishman et al has shown this year that spinal manipulative procedures lead to an increase in central motor excitability rather than overall inhibition. Specifically, their research report and their review of the literature showed that there is a postsynaptic facilitation of α motoneurons and/or corticomotoneurons that may be unique to the chiropractic spinal manipulative thrust [56].

The use of CMT for the correction of motor deficits found in symptomatic patients is the rationale for most of the systems of manual therapy that employ the MMT [1,5].

These studies support the concept that a close relationship and mutual influence exist between joints, soft tissue, muscles and the nervous system. Neglect of any one of these areas may lessen our diagnostic as well as therapeutic possibilities. For this reason the addition of the MMT into standard chiropractic diagnostic methods for the diagnosis of muscle inhibitions may be useful.

According to Janda [33], the four most typical types of functional muscle weakness that may be detected with the MMT are as follows:

**1. ***Tightness weakness *develops when a muscle is chronically shortened and eventually loses strength. Janda has reported that even when a muscle appears to be tight or stiff, some decrease in muscle strength occurs. Brooks confirms that chronically contracted muscles are weaker than muscles with a normal length [57]. Leahy says it simply: "When a muscle is tight it tends to weaken and when a muscle is weak it tends to be tight" [58].

**2. ***Stretch weakness *occurs if a muscle is perpetually placed in a lengthened position so that the muscle spindles become desensitized to stretch [59].

**3. ***Arthrogenic weakness *occurs when nociceptive afferent barrage from a joint or ligament causes reflex inhibition. Examples include the vastus medialis after injury of the anterior cruciate ligament or meniscus, or gluteus maximus weakness when a sacroiliac dysfunction is present [38-43,59,60].

**4. **Finally, *trigger point weakness *occurs when a muscle cannot fully activate all its contractile fibers because of the presence of a trigger point. Headley and Simons both report muscle inhibition during movement when trigger points are present [61,62].

These data indicate that the body's reaction to injury and pain is not primarily increased muscular tension and stiffness; rather *muscle inhibition is often more significant *[31,32,63]. Because of Sherrington's Law of Reciprocal Inhibition, these two functional states in muscles are related [64]. Sherrington's law states that decreased activity of certain muscles leads to facilitation – and thus increased activity and tension – of their antagonist muscles.

Lund suggests that the pain-spasm-pain model should be overturned and replaced with the pain-adaptation model to explain these muscle weaknesses [65]. He reviewed articles describing motor function in five chronic musculoskeletal pain conditions (temporomandibular disorders, muscle tension headache, fibromyalgia, chronic lower back pain, and post-exercise muscle soreness). Lund shows that when pain is present in each of these musculoskeletal disorders, there is a *decreased *activation of muscles during movements in which they act as agonists and increased activation during movements in which they are antagonists. This model is in clear contrast to the pain-spasm-pain model, which suggests that muscle tension is necessarily *increased *when painful stimuli are present.

Edgerton et al. found specifically that underactivity of agonist muscles and overactivity of synergist muscles were able to discriminate chronic neck pain patients due to whiplash injuries from those who had recovered with 88% accuracy [48]. Other research papers on whiplash-associated disorders have shown this pattern as well, in which inhibition of the deep neck flexor muscles will persist for some time after the injury [45].

An important diagnostic parameter of spinal dysfunction has been range of motion impairments. Muscle weakness may cause a loss of movement in the sense that a muscle cannot contract sufficiently to move the part through its complete range of motion [66]. When there is restriction of joint motion because of muscle spasm, the differentiation of whether muscle inhibition or muscle spasm is producing the restricted range of motion must be determined. The MMT is one method for making this determination.

Another reason for the addition of the MMT to other established methods of chiropractic diagnosis is that the MMT provides information about the patient that we did not already know. In a typical chiropractic clinical encounter, a patient comes for care because of musculoskeletal pain. The doctor performs a battery of tests that reproduce the pain, and he is therefore determined to have musculoskeletal pain. This is a somewhat circular process.

Where diagnostic methods have a capacity to specify the form of therapy needed or the prognosis or long-term course of a disorder, the diagnosis has increased value. This diagnostic value of MMT is characterized by using MMT to identify a functional disorder (inhibition) of the locomotor system, as well as the chiropractic manipulative treatment (CMT) to correct the findings of the inhibited MMT. The MMT diagnosis of inhibited muscles and their covariance with patients' musculoskeletal dysfunctions may be able to tell us something about the status of their condition as well as the responsiveness of this musculoskeletal disorder to treatment. The immediate improvement in muscle strength and its covariance with patients' dysfunctions after CMT that has been reported clinically suggests this correlation as well [49-56,67].

If a patient's radicular pain peripheralizes, research has suggested that their condition is worsening [68]. If a patient's muscle strength weakens, this likewise indicates that their condition is worsening. Assessing the function of muscles with the MMT pre- and post-treatment is hypothesized to assess the effects of a therapeutic intervention aimed at improving muscle performance. This assessment process is the basis of the chiropractic use of the MMT.

Muscle weakness commonly indicates neurological and/or orthopedic changes in the joint, muscles, or nerve supply [1,2,10-16]. If the patient has increased strength during the course of treatment, immediately or over time, this would be considered a positive result as well.

Patients want to know what is causing their disorder. Although a functional MMT does not pinpoint causality it does give the clinician and the patient targets for functional reactivation as well as providing inexpensive and reliable tests that can be used to audit the patient's status and his progress toward functional restoration.

A final reason for the addition of MMT to chiropractic diagnostic methods is that most other parameters of dysfunction identified in low-back and neck pain patients have not been shown to precede the pain, but rather only to accompany it. An important exception is muscle strength, which can predict future low-back and neck pain in asymptomatic individuals [48,69-72].

Published studies suggest that new methods of management are required to tackle the growing prevalence of spinal and spinal-related pain in society [73]. A new assessment protocol that may help diagnose neuromusculoskeletal dysfunction before it becomes chronic could significantly aid health care practitioners. These additional methods of diagnosis are needed because traditional examination methods such as neurologic, orthopedic, and imaging tests are able to accurately diagnose the cause of pain in only some 10% of patients [74]. The use of the MMT for the diagnosis of musculoskeletal dysfunction has already been accepted as valid by the medical, physical therapy, neurology and other professional health care communities. The system of MMT used in AK (based on the works of Kendall & Kendall) has been accepted by the American Medical Association in its *Guides to the Evaluation of Permanent Impairment*, 5th edition, as a reliable and valid method for evaluating functional, non-pathological, radicular, and non-radicular neuromusculoskeletal conditions [75].

## Clinical guidelines for the manual muscle test

The representative techniques of MMT presented here are based on the work of a number of investigators. No attempt will be made to present all the tests devised for any particular muscle. Instead, nine important parameters of the MMT procedure that should be followed when testing any muscle will be presented in order to attain reliability and validity with this diagnostic tool.

**1. **Is the test used a standardized MMT of the muscle or group of muscles, or is it a general test such as 'the arm test'?

**2. **On how many muscles is the procedure valid?

**3. **Are the starting point and the direction of force the same each time the muscle is tested?

**4. **Does the tester apply the same force with the same timing each time the muscle is tested, i.e. does the tester apply the force to the patient at a constant rate and speed?

**5. **Is the contact point on the patient the same each time the muscle is tested?

**6. **Is the tester's hand contact with the patient the same each time the muscle is tested?

**7. **Are the tester's elbow, arm and forearm in the same position for each test?

**8. **Are the tester's shoulders relaxed and in the same plane each time the muscle is tested?

**9. **Is the tester's body in the same position with the core muscles of his body engaged in the same way each time he tests the muscle?

An explanation for each of these clinical guidelines follows:

### 1. Is the test used a standardized MMT of the muscle or group of muscles, or is it a general test such as 'the arm test'?

Much error in muscle testing is a result of testing a general group of muscles rather than a specific muscle. General tests such as "the arm test" are actually, at best, testing a gait function, a series of muscles, rather than a specific muscle. The type of response gathered from the MMT depends on the type of MMT employed, and "the arm test" gives a different response than do the standardized tests of specific muscles.

The standard references for muscle testing evaluation as accepted by the I.C.A.K. are the original work of Kendall and Kendall, *Muscles: Testing and Function *[12], and the modifications suggested by Goodheart in his *Applied Kinesiology Research Manuals *[1]. Goodheart's and the I.C.A.K.'s investigations into the use of the MMT for chiropractic diagnosis have been well organized and disseminated to the professions by Walther and others [7,15,16,76-78].

It is critical that the MMT protocol be highly reproducible by the examiner and by others. The earliest books on the use of the MMT for the functional assessment of patients argue that appropriate methodological techniques must be strictly followed before obtaining or interpreting MMT outcomes [1,10-13]. This call still echoes among the numerous abuses that have been promulgated throughout the past 40 years of MMT use in the manipulative professions [18-25,27].

**Figure 1:**
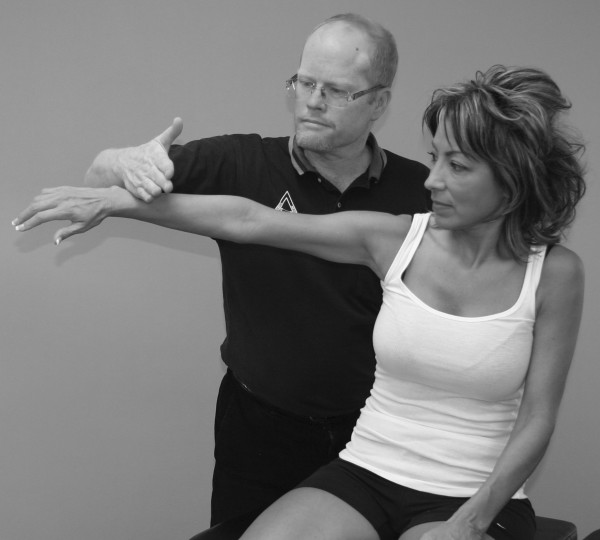
**The "arm test" does not isolate nor specifically test any particular shoulder muscle**.

**Figure 2:**
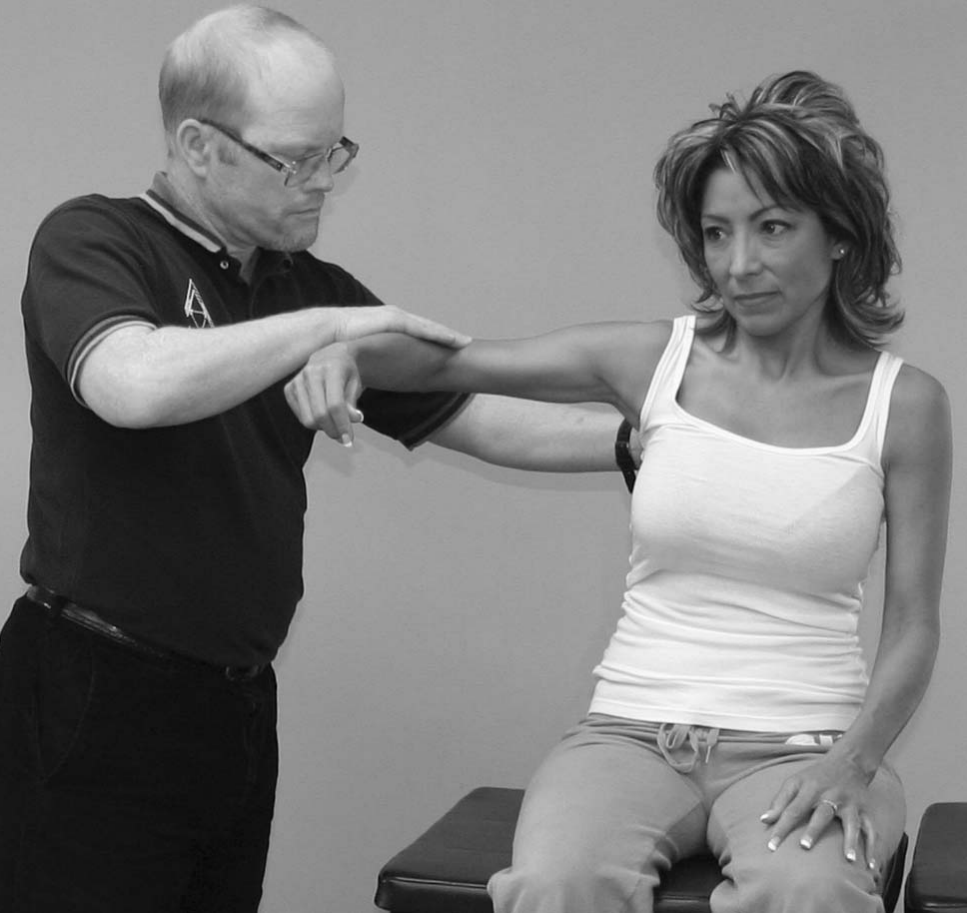
**Middle deltoid MMT**.

**Figure 3:**
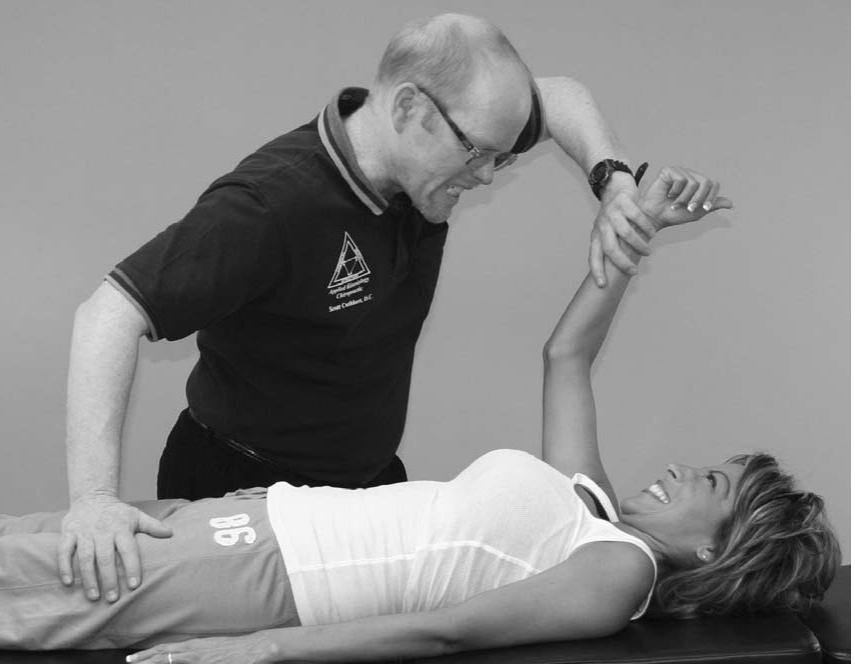
**The MMT is not a competition between the examiner and the patient**.

An understanding of the principles in the original works of Kendall, Goodheart, and Walther is necessary for using the MMT. The testing procedures from these volumes may be modified slightly, depending on the structure of the patient, but must be consistent from test to test on the same individual. Observe the difference between the two tests shown in Figures 1 and 2. Figure 1 shows "the arm test" while Figure 2 shows the middle deltoid MMT. "The arm test" monitors all the arm flexors and abductors as a group, and the middle deltoid isolates a specific muscle and evaluates the neurological functions thereby identified. In figure 1 the patient's head is also turned and she is leaning her torso onto her left hip. Figure 3 shows that the MMT is not a contest between the patient and the doctor.

It should be observed that a relationship between shoulder pain and dysfunction and specific muscle weakness has been established in a number of studies [79-84].

The MMT should evaluate individual muscles as far as possible. There is an overlap of muscle actions, as well as an interdependence of muscles in movement. This close relationship in muscle function need not rule out the possibility or the practicability of testing individual muscles. There is an ideal starting position and vector of testing force that places the muscle being tested as the prime mover and the synergists at a disadvantage during the test.

**Figure 4:**
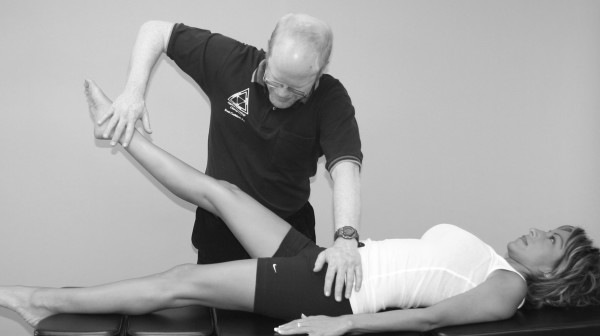
**MMT of the psoas major muscle**. It shows that the quadriceps, sartorius, and adductor muscles all assist in holding the hip in a flexion position. However, the line of pull of the muscle and the direction of the examiner's pressure place emphasis on the action of the right psoas major, making identification of inhibition in this specific muscle possible.

Janda (who also used the MMT to evaluate locomotor dysfunction) has emphasized that prime movers and synergists are tested with the MMT, not individual muscles [63]. However, it should be pointed out that every muscle is a prime mover in some specific action. In the search for that action, one is led into the field of precise, individual muscle testing. Manual muscle tests are designed to replicate the primary vector of motion of a muscle while minimizing the contribution of secondary mover muscles. During an individual MMT, the designated primary mover muscle should have the highest level of activity compared with the secondary mover or synergist muscles. When any one muscle in the body is inhibited in its strength or action, stability of the part is impaired or some exact movement is lost to some extent. When inhibition of a muscle results in the inability to hold the test position or perform the test movement ascribed to that muscle, the validity of the individual muscle test is substantiated (Figure 4).

### 2. On how many muscles is the procedure valid?

**Figure 5:**
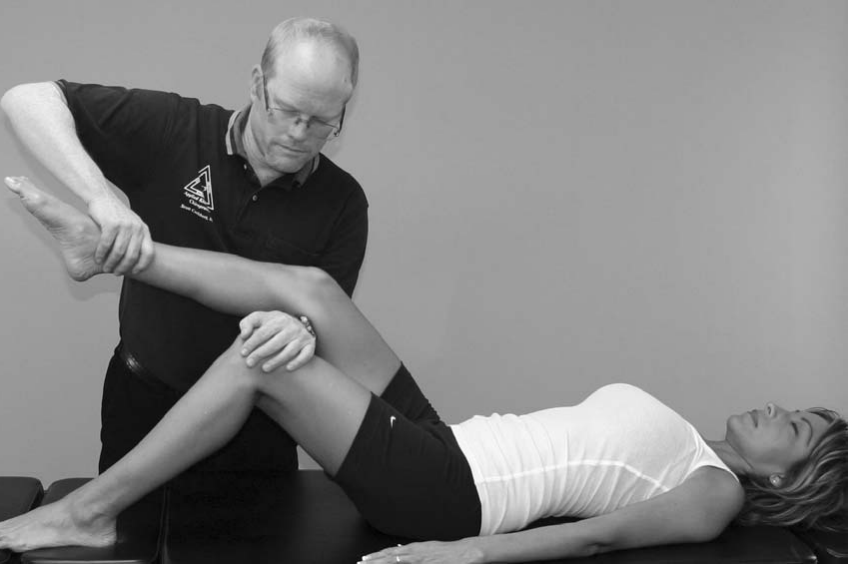
**Quadriceps femoris MMT**.

The Research Committee of the I.C.A.K. has adopted a policy wherein any new diagnostic or manipulative treatment technique must be evaluated using three separate and distinct muscles, one of which is the quadriceps femoris tested in the supine position (Figure 5), before it is considered reproducible and valid. Many times we see a technique or research paper presented using "the arm test" (which is easily misperformed or misinterpreted) that cannot be reproduced when applied to another muscle, especially the large and powerful quadriceps femoris muscle [18,20,21].

It should be observed that a relationship between knee pain and dysfunction and muscle weakness has been identified in a number of studies as well [38-40,84-88].

### 3. Are the starting point and the direction of force the same each time the muscle is tested?

**Figure 6:**
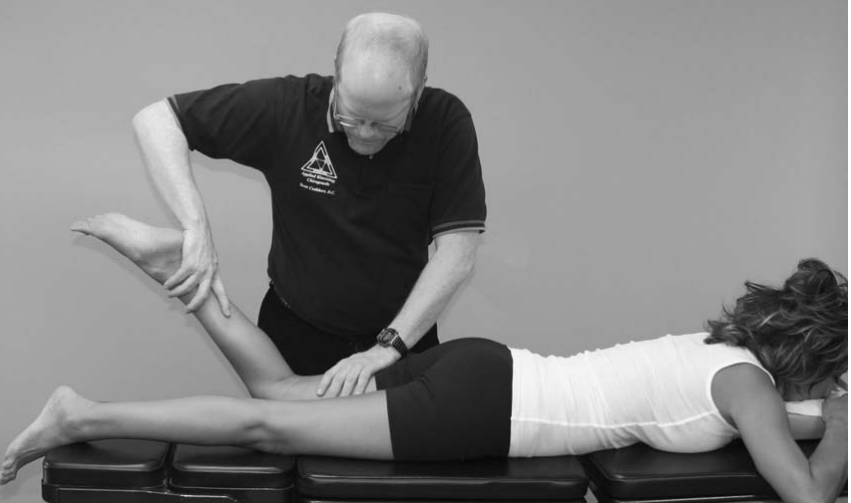
**Hamstring MMT**.

**Figure 7:**
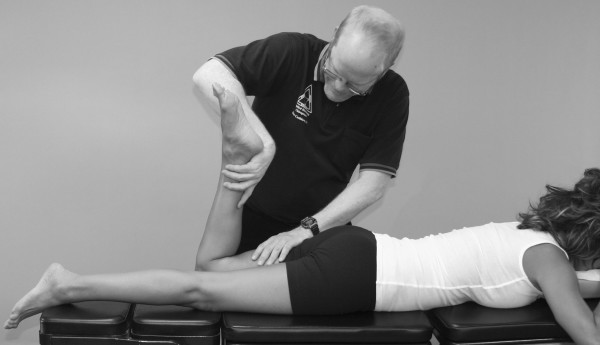
**Hamstring MMT incorrectly done**. Knee excessively flexed allows muscles to cramp and makes the test difficult to judge.

The enthusiasm for a new idea has many times blinded the tester from realizing that he alters the starting position of the test and his line of force. From one test to the next this may vary as much as several inches or 45 degrees, thereby invalidating the data he receives from the test. The starting point should be consistent. The line of force should not vary more than a few degrees from test to test. Failure to strictly follow these guidelines leads to substitution of synergistic muscle function replacing or supplanting the muscle that is being examined, thereby altering the parameter being examined (Figures 6 and 7).

Poor motor control – as demonstrated by synergist substitution that must be carefully monitored and prevented during the MMT – has been linked to decreased joint stability [48,89,90]. As mentioned previously, Lund hypothesizes that when pain is present, there is decreased activation of muscles during movements in which they act as agonists and increased activation during movements in which they are antagonists. Rather it appears that muscle imbalance is the rule in injuries, pain, and inflammation, with certain muscles tending toward inhibition and others toward hyperactivity. This explanation is more in line with the common impression that pain makes muscles difficult to use and less powerful [91].

Synergist substitution may be the body's attempt to compensate for an inhibited muscle that is not adequately stabilizing a joint. Edgerton reports that synergist substitution for inhibited muscles distinguished chronic neck pain patients from asymptomatic patients after whiplash injury [48]. In these patients, overall muscle strength may not be inhibited if tested with a dynamometer because synergists substitute for the specific inhibited agonist muscles that should be identified by precise positioning during the MMT.

For accurate MMT examination, no substitutions should be permitted. The position or movement described as the MMT should be done without shifting the body or turning the part to allow other muscles to substitute for the weak muscle. It is natural for the subject to change the MMT parameters to recruit synergistic muscles in the presence of a weak prime mover. Accurate MMT depends upon the examiner's awareness of this factor and the ability to detect it when it occurs. Because synergist-agonist substitution for inhibited muscles is so common in neuromusculoskeletal dysfunction [65,66]. the importance of specific (not group) MMT is once again apparent.

**Figure 8:**
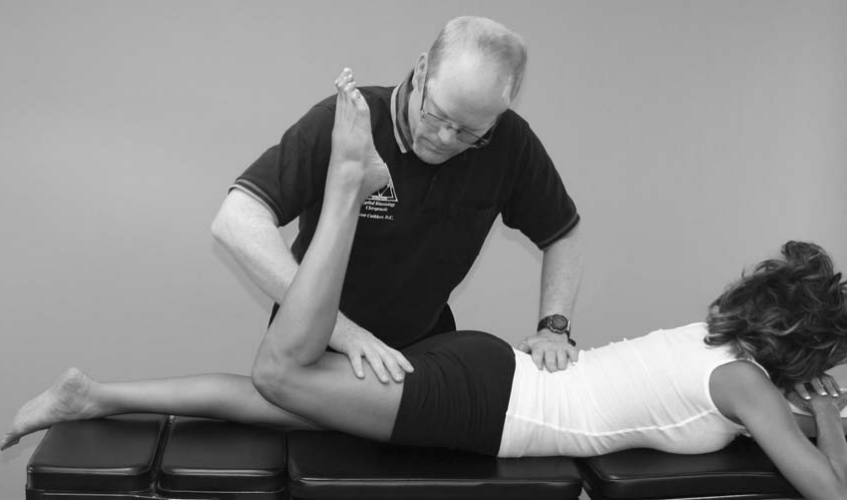
**Gluteus Maximus MMT**.

**Figure 9:**
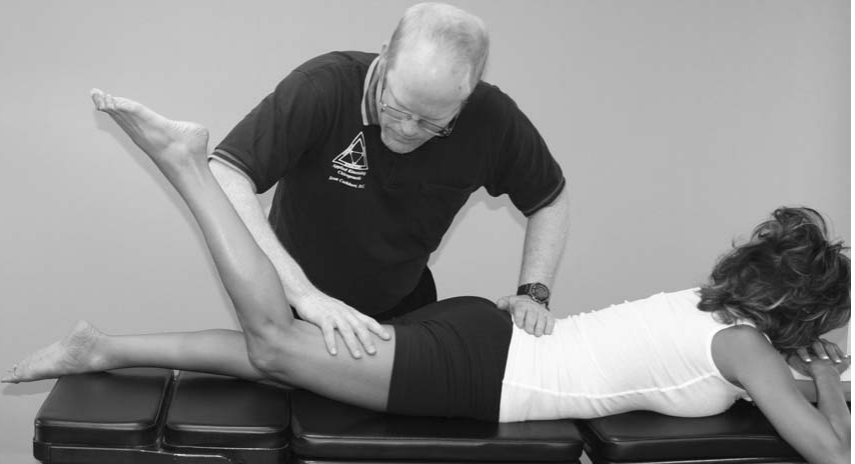
**Gluteus Maximus test incorrectly done: excessive extension**. Patient tends to straighten leg to recruit more hamstring synergism. Knee flexion helps eliminate the hamstring's contribution to the test.

**Figure 10:**
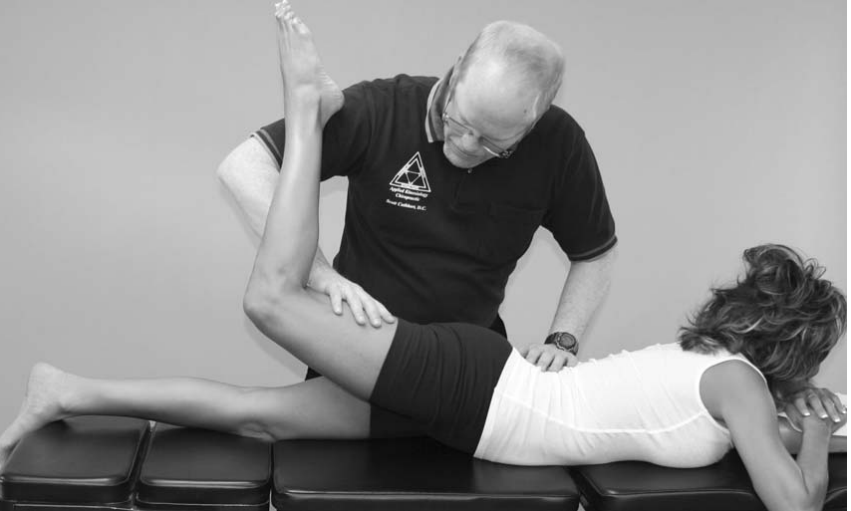
**Synergist substitution can be identified and prevented during the MMT**. With a weak gluteus maximus, the examiner can visualize a lifting of the pelvis with external rotation and abduction of the hip, with recruitment of the ipsilateral hamstring, thoracolumbar extensors, and contralateral leg flexor muscles. The pelvis externally rotates because the weak gluteus maximus recruits synergists to facilitate its action during the MMT.

Synergist substitution is frequently seen in impairments of gluteus maximus function on the MMT [2,15,59,66]. It should also be observed that a relationship between low-back dysfunction and pain and specific muscle weakness in the gluteus maximus muscle has been established in a number of studies (Figures 8, 9 and 10) [43,90,92].

### 4. Does the tester apply the force to the patient at a constant rate, i.e. does the tester apply the same force each time the muscle is tested?

It is easy to overpower even the strongest patient if you apply force too rapidly or "jump the gun" as it is often referred to. Muscle testing evaluates the *strength *of response of the muscle, not the *speed *of response. Muscle testing is an art in which the force applied to the patient is increased at a constant rate until the tester senses the muscle *begin *to give way. The classic "break test" used by physical therapists tests this phenomenon as well [11-13]. Clinically this is then compared with the amount of force needed to cause the muscle to begin to give way following the application of a variety of treatment and assessment procedures, and the tester must accurately monitor whether or not there is a difference.

In presenting MMT and AK methods to an audience, many of these subtleties are not easily conveyed. This leads the lecturer to test the muscle through its entire range of motion in order to bring the point across to the audience. It is not, however, the recommended practice for clinical use. As Walther states, "Once the muscle is in motion, the test is over" [15]. The amount of force required to initiate motion is the parameter that should be measured in accurate MMT. Overpowering a muscle can be noted when the tester applies the force too rapidly or forces the muscle through its entire range of motion before determining its ability to resist.

A previous literature review in this journal [93] as well as other research reports has shown the importance of clinical experience and expertise concerning the reliability of the MMT [11-13]. The skills of the examiners conducting studies on MMT and their skills in interpreting the derived information will affect the usefulness of MMT data. Examiners are obliged to follow standardized protocols that specify examiner and patient position, the precise alignment of the muscle being tested, the direction of the resisting force applied to the patient, and the verbal instruction or demonstration to the patient.

An experienced examiner who is aware of the ease with which normal muscles perform the MMT will readily detect substitutions if there is weakness. Even an inexperienced examiner can often detect the sudden shift of the body that results from the effort to compensate for muscle weakness during the MMT.

Mendell and Florence (1990) [94], Caruso and Leisman (2000) [95], and other researchers of MMT have discussed the importance of considering the examiner's training for the interpretation of studies that assess strength via MMT.

From these studies it appears obvious that training and skill are necessary to perform these tests properly and to interpret their outcomes reliably. MMT for functional neuromusculoskeletal evaluation is more sophisticated than simply asking the patient to shrug the shoulders to ascertain if cranial nerve XI is intact. When conducted properly the procedures have reported significant inter- and intra-examiner reliability as well as significant construct, content, concurrent and predictive validity [93].

### 5. Is the contact point on the patient the same each time the muscle is tested?

The point of contact between the tester and the patient can be a critical factor for two reasons. First, the amount of leverage the tester has at his advantage can alter the performance of the test. The contact point of the tester's hand on the patient should not vary more than 1/2 inch from test to test.

Second, many areas of the body are extremely sensitive to pressure; thus a patient's muscle may yield not to the force put on it, but to the pain from the tester's contact point. This is especially true of the wrist and ankle, where the bone is very sensitive and not adequately padded by soft tissue.

**Figure 11:**
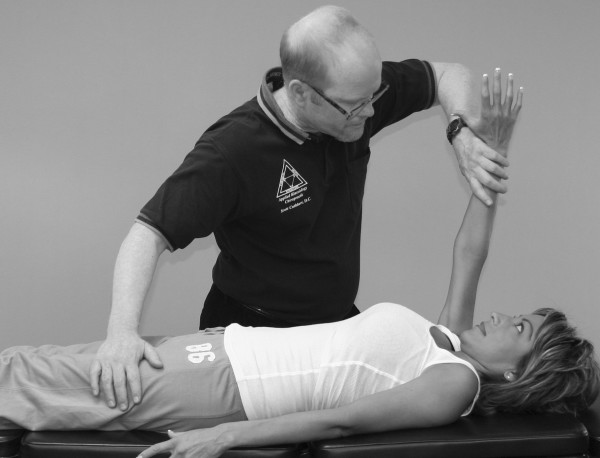
**Pectoralis major muscle (sternal division), proper hand contact**.

**Figure 12:**
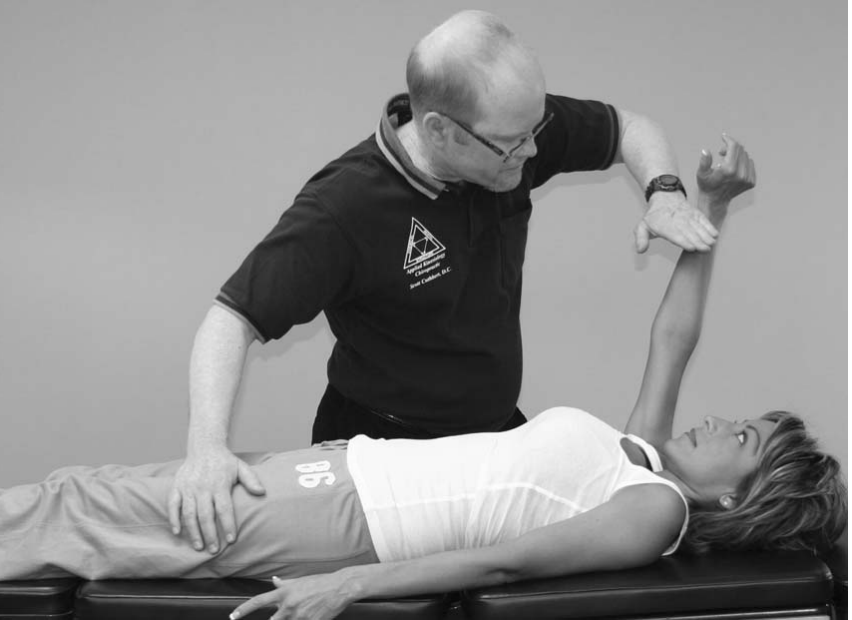
**Hand position changed, painful contact on bony prominence**.

Many tests also require that the tester provide stabilization for the patient with the hand other than the testing hand. The stabilization hand should be placed in the same position every time the muscle is tested. It is very easy for the over-enthusiastic tester to properly stabilize the patient on one test and to unknowingly allow the previously stabilized body part to move on subsequent tests. In the case of normally strong pectoralis major (sternal division), or psoas major muscles, lack of proper stabilization may cause the muscles to appear weak because the patient allows them to give way when he feels his body beginning to fall off the table. The tested muscle must always be functioning from a stable base during MMT. Care must also be taken to ensure that the position of the stabilizing hand on the patient does not cause pain, which would again cause him to release his resistance (Figures 11 and 12).

### 6. Is the tester's hand contact with the patient the same each time the muscle is tested?

**Figure 13:**
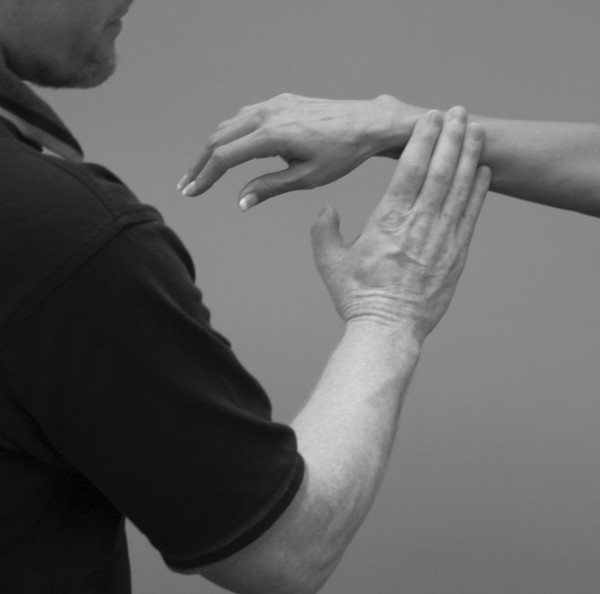
**Hand contact – fingertips**.

**Figure 14:**
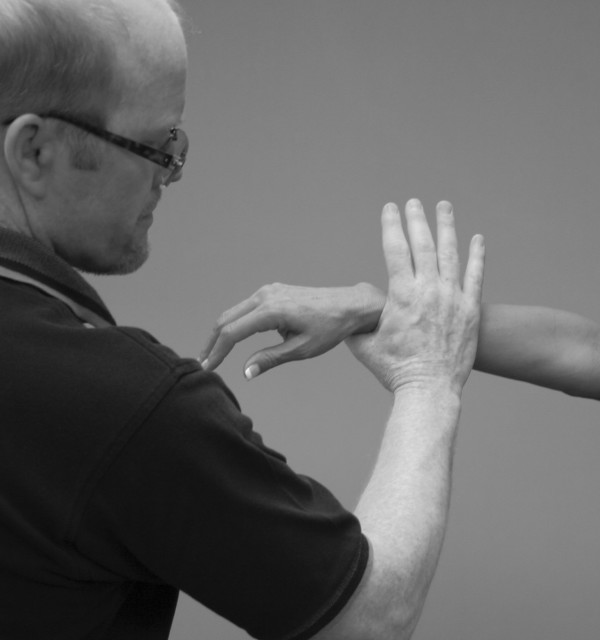
**Hand contact – full palm**.

This is a very important and often overlooked criterion. Notice the difference between the part of the hand with which the tester applies pressure in Figures 13 and 14. Proper muscle testing involves the sensitivity of touch-pressure and joint receptors in the examiner's fingers and hands. Proper discrimination in the amount of force applied must be monitored by the examiner's fingers. Hence the examiner must keep his awareness primarily centered on the amount of pressure he senses through his fingers, and to a lesser extent, his wrist, elbow and shoulders. The MMT with the fingers on one test and then with the palm on another test will cause one to interpret the finger test as stronger than the palm test since the brain receives more impulses from the rich endowment of nerve endings of the fingers, regardless of the actual force exerted. An examiner who is not cognizant of this fact may inadvertently change the area of his hand that contacts the patient from test to test, and his brain will interpret and process the disparate information which it receives. This is a critical area that allows many examiners to deceive themselves, only to become embarrassed at a later date when they discover what they are actually doing.

### 7. Are the tester's elbow, arm and forearm in the same position for each test?

**Figure 15:**
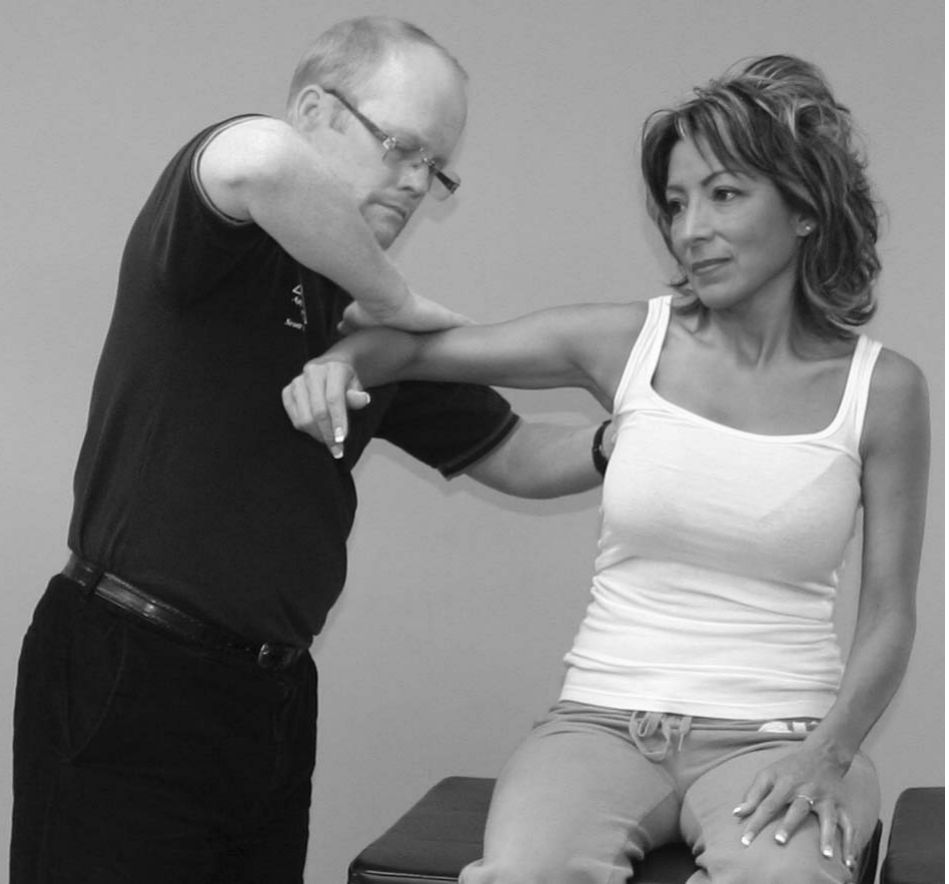
**Middle deltoid MMT – mechanical advantage**.

Note the difference in the examiner's elbow, arm and forearm positions in Figures 2 and 15. One can readily see the difference in leverage the examiner exerts at each position. Note in Figure 15 that the examiner has a tendency to push down with the weight of his arm (and possibly his whole body) rather than exert pressure through his fingers as discussed above (and shown in Figure 2). Also notice in Figure 15 that the examiner has the tendency to try to force his entire body weight on the patient's arm, thus overpowering her. These errors in testing are sometimes due to the disparity in height between the examiner and patient. In both cases, the examiner has the tendency to judge the amount of pressure he exerts not by the finger receptors as discussed above, but by the wrist, elbow, and shoulder proprioceptors. This method yields inconsistent and therefore invalid results. The arm, forearm and elbow positions should be the same each time the test is performed. Kendall, Walther, and others have extensively described the clinical guidelines for doctor and patient positioning during the MMT for each muscle, and this training is available through several of the chiropractic colleges and the I.C.A.K [1,12,13,15,96].

### 8. Are the tester's shoulders relaxed and in the same plane each time the muscle is tested?

**Figure 16:**
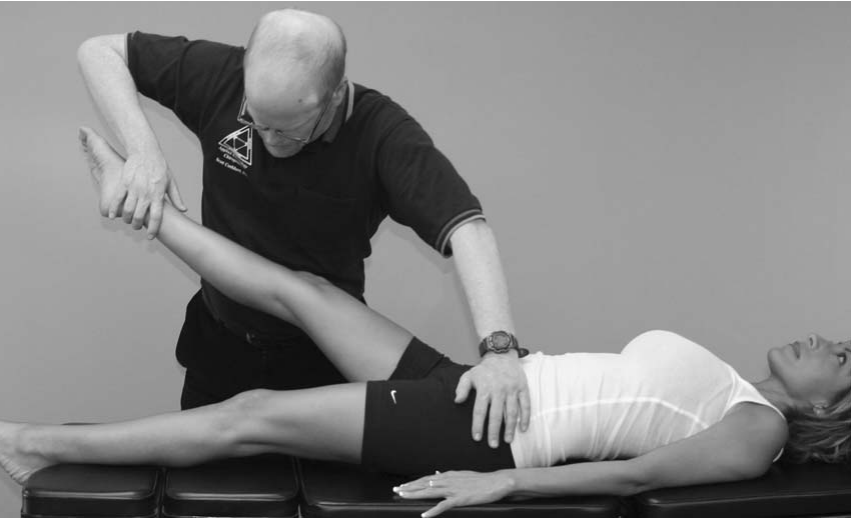
**MMT of the psoas major muscle-mechanical advantage**.

Compare the level of the shoulders in Figures 2 and 15. Figure 4 shows the psoas major MMT being performed properly. Figure 16 shows the examiner leaning over the patient and inadvertently transferring his entire body weight to the patient's leg. This is a very common error observed in undertrained muscle testers.

### 9. Is the tester's body in the same position with the core muscles of his body engaged in the same way each time he tests the muscle?

**Figure 17:**
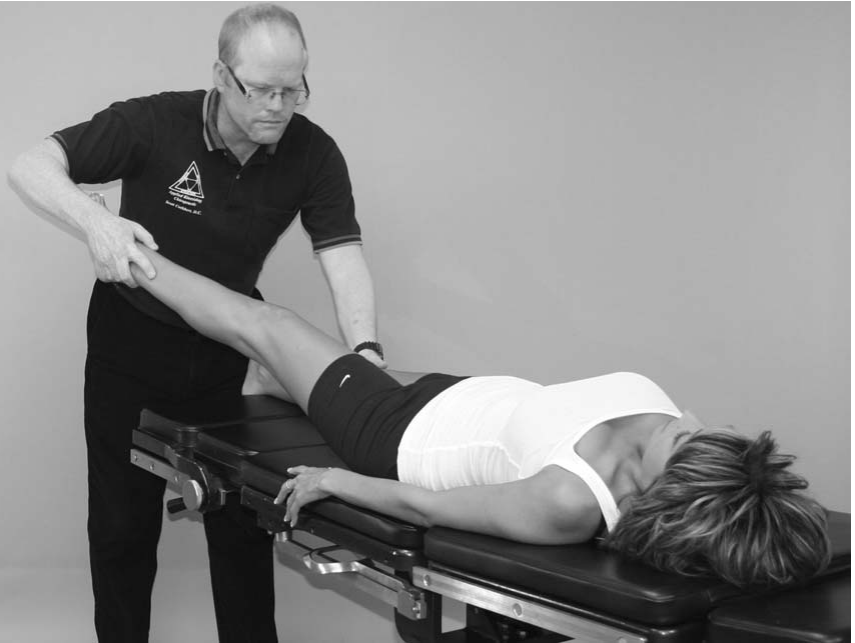
**Tensor fascia lata MMT**.

This error in muscle testing, most often associated with "the arm test", involves the examiner literally leaning his entire body weight on the patient. This is demonstrated by the difference between Figures 2 (normal) and 15 (leaning) and Figures 17 (normal) and 18 (leaning). This mistake can be avoided if the examiner places his feet and his umbilicus in the same position each time he tests the patient.

**Figure 18:**
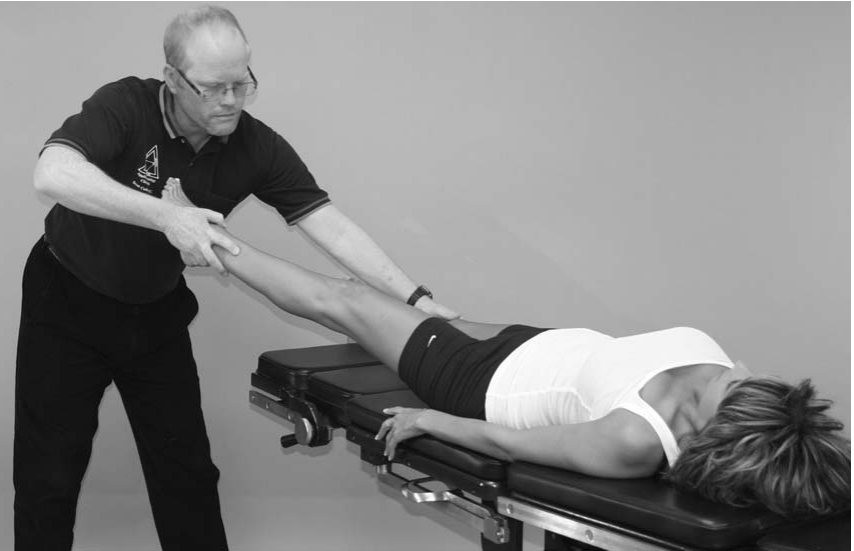
**Examiner changes position during tensor fascia lata MMT**.

## Conclusion

The addition of the MMT to chiropractic diagnostic methods has generated interest in these procedures from many disciplines of the healing arts. Muscle testing as a measurement of the functional status of the neuromuscular system has offered additional diagnostic parameters for clinical research in the assessment of patients with physical dysfunctions that are treated by chiropractors, orthopedists, dentists, physical therapists, osteopaths, and general medical physicians. The MMT may enhance clinical decision-making and lead to better patient care through detection of change or lack of change in the patient's motor performance after manipulative treatment.

This paper aims to heighten the importance and awareness of accuracy when using the MMT as an examination tool. It presents some of the most common mistakes that have been adopted in the use of the MMT in clinical and research settings (Table 1).

**Table 1:** Summary recommendations for manual muscle testing.

**1. **Is the test used a standardized MMT of the muscle or group of muscles, or is it a general test, such as "the arm test" which does not specifically test the strength of any one muscle.
**2. **On how many muscles is the procedure valid?

**3. **Are the starting point and the direction of force the same each time the muscle is tested?

**4. **Does the tester apply the same force each time the muscle is tested, i.e. does the tester apply the force to the patient at a constant rate?

**5. **Is the contact point on the patient the same each time the muscle is tested?

**6. **Is the tester's hand contact with the patient the same each time the muscle is tested?

**7. **Are the tester's elbow, arm and forearm in the same position for each test?

**8. **Are the tester's shoulders relaxed and in the same plane each time the muscle is tested?

**9. **Is the tester's body in the same position with the core muscles of his body engaged in the same way each time he tests the muscle?

The original method for testing muscles and determining their functional state, first advocated by Kendall and Kendall and then applied to chiropractic methods by Goodheart, is a diagnostic device whose potential will not be realized until this tool is used precisely. While Goodheart developed many testing refinements and new hypotheses for the MMT that will require more controlled clinical trials to test their validity, and the usefulness of the MMT in diagnosing all of the physiologic conditions it is currently used for will require more substantiation. Appropriately selecting and clustering patients for controlled clinical trials to evaluate this method will depend upon the application of accurate and reliable procedures of MMT.

The use of the clinical guidelines for the MMT as described in this paper is primarily for the investigation of neuromusculoskeletal dysfunction, rather than for many of the other less investigated uses of the MMT. The MMT that measures muscle performance provides unique impairment information for determining diagnosis, prognosis, and plan of care for patients with neuromusculoskeletal dysfunctions. There is no other method available in the clinical setting for testing muscle strength and function that is as reliable, easy-to-use, inexpensive, non-invasive and possessing the "face-validity" as the MMT. Moreover these tests can be used to assess the effects of interventions aimed at improving muscle performance.

This paper is not intended to discredit anyone who is using the MMT clinically or for experimental investigations in clinical or research settings. On the contrary, it is hoped that this paper may assist in the self-appraisal and peer-appraisal for all those using the MMT as a parameter of clinical investigation and for the eradication of operator prejudice during these procedures.

## Competing interests

WHS is a diplomate of the International College of Applied Kinesiology (I.C.A.K-USA). SCC is a Board Member for the I.C.A.K.-USA. SCC and WHS both employ MMT and AK methods in their evaluation and treatment of patients.

## Authors' contributions

WHS and SCC conceived the research idea. SCC constructed the literature review. SCC and WHS drafted the manuscript and approved the final version for publication.
